# Photonic Crystal Flip-Flops: Recent Developments in All Optical Memory Components

**DOI:** 10.3390/ma16196467

**Published:** 2023-09-28

**Authors:** Yonatan Pugachov, Moria Gulitski, Dror Malka

**Affiliations:** Faculty of Engineering, Holon Institute of Technology (HIT), Holon 5810201, Israel; yoni.pugachov@gmail.com (Y.P.); moriagulitski@gmail.com (M.G.)

**Keywords:** photonic crystals, flip flop, optical memory, FDTD, PWE, optical computing, optical communication

## Abstract

This paper reviews recent advancements in all-optical memory components, particularly focusing on various types of all-optical flip-flops (FFs) based on photonic crystal (PC) structures proposed in recent years. PCs, with their unique optical properties and engineered structures, including photonic bandgap control, enhanced light–matter interaction, and compact size, make them especially suitable for optical FFs. The study explores three key materials, silicon, chalcogenide glass, and gallium arsenide, known for their high refractive index contrast, compact size, hybrid integration capability, and easy fabrication processes. Furthermore, these materials exhibit excellent compatibility with different technologies like CMOS and fiber optics, enhancing their versatility in various applications. The structures proposed in the research leverage mechanisms such as waveguides, ring resonators, scattering rods, coupling rods, edge rods, switches, resonant cavities, and multi-mode interference. The paper delves into crucial properties and parameters of all-optical FFs, including response time, contrast ratio, and operating wavelength. Optical FFs possess significant advantages, such as high speed, low power consumption, and potential for integration, making them a promising technology for advancing optical computing and optical memory systems.

## 1. Introduction

Traditional electronic memory devices face physical and electrical limitations in terms of speed, power consumption, and integration density, necessitating the exploration of alternative technologies. Therefore, optical computing serves as a viable alternative to conventional electrical memory devices. In the rapidly evolving field of optical computing and information processing, the demand for high-speed and low-power memory components has been a driving force behind ongoing research and development efforts.

Computer memory is the ability of a system to store, retain, and recall data or instructions. It is a crucial component that enables computers and other electronic devices to perform various tasks efficiently. Memory is categorized into different types based on its characteristics, speed, capacity, and purpose. Of the two primary types of memory used in computing, Random Access Memory (RAM) is the main memory of a computer that provides fast and temporary storage for data and program instructions. It allows the CPU to access data quickly, enabling the rapid execution of tasks. RAM is volatile, which means its contents are lost when the power is turned off. Read-Only Memory (ROM) is a type of memory that stores permanent data and instructions necessary for the basic functioning of a computer or device. Unlike RAM, ROM is non-volatile, meaning its contents remain intact even when the power is off.

Computer memory systems utilize multiple flip-flops (FFs) arranged in arrays or hierarchical structures to create larger memory units. This allows for the storage and retrieval of data systematically and efficiently, in addition to more extensive data storage and retrieval capabilities. The arrangement and organization of FFs determine the type of memory architecture, such as RAM [1,2] or ROM [3].

FFs [4,5,6] serve as memory components by storing and retaining binary information. Each FF can store one bit of data, representing either a 0 or a 1. The state of the FF is determined by its inputs and the clock (CLK) signal. When an FF receives a CLK signal, it captures the input data and holds them until the next CLK cycle. The captured data become the output of the FF. These data remain stored in the FF even when the inputs change or the CLK signal stops. The stored data in FFs can be read and used by other logic circuits or fed back as inputs to the FFs themselves, enabling the creation of sequential logic circuits. The ability of FFs to maintain their state until explicitly changed by an input or CLK signal makes them suitable for implementing memory elements in digital systems, such as in shift registers or memory arrays.

The memory unit in a computer is a crucial component responsible for storing and retrieving data and instructions. It provides temporary storage for the computer’s operating system, software applications, and user data during program execution. Additionally, the memory unit serves as a working space for the processor, allowing for fast and efficient access to the information needed for processing. Given its integration with other components, power consumption, dimensions, and operational speed are crucial considerations that significantly impact system performance. These factors significantly affect the overall performance and functionality of the system.

Optical technologies offer opportunities for achieving high-speed, low-power, and small dimensions when implementing memory systems. Optical memories can be applied in various applications and systems where high-speed and high-capacity data storage is required; for instance, optical routing [7,8,9], optical switching [10,11], video surveillance systems [12,13], optical fiber [14,15,16], and optical interconnects for high-performance computing [17,18].

Photonic crystals (PCs) [19,20], with their unique optical properties and engineered structures, have emerged as promising candidates for the realization of efficient and compact all-optical memory components. Their ability to manipulate light at the nanoscale offers promising opportunities for advanced data storage and processing in optical memory technologies. These unique properties of PC-engineered structures have led to the design and fabrication of various elements such as power splitters [21,22], power combiners [23], resonators [24,25], modulators [26,27], couplers [28,29], interferometers [30,31], switches [32], logic gates [33,34], dividers [35,36], sensors [37,38,39], filters [40], analog-to-digital converters [41,42], multiplexers [43], and demultiplexers [44].

The paper examines three main materials: silicon, chalcogenide glass, and gallium arsenide, all of which possess excellent attributes for constructing all-optical memory components. It is important to consider additional materials like silicon nitride [45], lithium niobate [46], and indium phosphide [47], as they exhibit low-loss properties that enable them to be used as a PC platform.

The development of all-optical memory components based on PC represents a significant advancement in the field of optical computing. These components have the potential to overcome the limitations of electronic memories, offering ultra-fast operation, low power consumption, and compatibility with high-speed optical data processing, by harnessing the unique properties of PC, such as enhanced light–matter interaction, slow light effects, and photonic bandgap (PBG) control.

Nonlinear effects [48] arise due to the nonlinearity in the polarization or refractive index of the material induced by intense light. When the intensity of light exceeds a certain threshold, these effects become significant. Some of the commonly observed nonlinear optical effects include the optical Kerr effect, which involves the change in the refractive index of a material in response to the intensity of incident light. This effect can lead to phenomena such as self-focusing or self-phase modulation, where the refractive index changes dynamically with the intensity of the light. The optical Kerr effect [49] is exploited in applications such as all-optical switching and signal processing.

PBG effects [50] refer to the control of light propagation through the creation of forbidden frequency ranges, known as bandgaps, in certain materials or structures. By exploiting PBG effects, specific wavelengths of light can be selectively controlled, stored, or retrieved in optical FFs.

Resonance effects [51] in PC occur when an optical system or device exhibits a strong response to a particular frequency of light. It happens when the frequency of an external light source matches the natural frequency of the system, resulting in a significant increase in the amplitude of the response. This resonance effect can enhance the interaction between light and matter, leading to efficient energy transfer, increased absorption, or stronger emission. The resonance effect is demonstrated in ring resonators (RR), enabling the selective enhancement and filtering of particular wavelengths of light.

Researchers have made significant strides in the design of PC FFs, which serve as key building blocks for all-optical memory systems. There are several types of FFs commonly used in digital electronics and sequential logic circuits. Some of the most widely used FF types include the following.

SR FF [52] (Set–Reset FF) has two inputs, set and reset, and two outputs, Q and Q’ (complementary output to Q). It can be in one of four states based on the input combinations, and it stores a single bit of data.

D FF [53] (Data FF) has a single data input D, a CLK input, and two outputs Q and Q’. The output Q follows the input D based on the CLK signal, capturing the input data at the rising or falling edge of the CLK.

JK FF [54] has two inputs J and K, a CLK input, and two outputs Q and Q’. It can be in one of four states and has additional functionality compared to the SR FF, including the ability to toggle or hold the output.

T FF [55] (Toggle FF) has a single input T, a CLK input, and two outputs Q and Q’. The output toggles (flips) its state when the input T is high and the CLK signal transitions.

These FFs serve different purposes in digital circuits and sequential logic designs, providing storage and memory functionality.

In the design of optical FFs, several optical effects are utilized to achieve the desired functionality. These effects leverage the unique properties of light and its interaction with materials. Some of the key optical effects used in designing optical FFs include the following.

The interference effect is typically most pronounced in a specific region of the waveguide structure known as the multi-mode interference (MMI) [56] region. In this region, the modes overlap and interact with each other, leading to constructive or destructive interference.

Resonant cavities in PC are regions within the crystal structure where light is confined and exhibits specific resonant modes. These cavities are formed by introducing defects or perturbations, also known as scattering rods, in the periodic arrangement of the PC, leading to localized field confinement and enhanced light–matter interactions.

All-optical switches [57] play a crucial role in controlling the flow of optical signals within optical FFs. All-optical switches are devices that enable the routing or switching of optical signals without the need for converting them into electronic signals.

Nonlinear optical switches [58] exploit the nonlinear properties of certain materials or components to achieve signal switching. One common approach is using the phenomenon of cross-phase modulation, wherein the refractive index of a material is modified by the intensity of an input optical signal. By controlling the intensity of the control signal, the transmission of the input signal can be modulated, effectively switching it on or off.

Some of the papers have used the Plane Wave Expansion (PWE) method, which is a powerful computational tool for analyzing the behavior of electromagnetic waves in periodic structures, for the PBG calculation, and Finite-Difference Time-Domain (FDTD), which is an effective numerical technique for simulating electromagnetic wave phenomena to achieve a simulation result.

This paper aims to provide a comprehensive overview of recent developments in PC FFs for all-optical memory components. We will discuss the fundamental principles underlying the operation of PC FFs and explore the design considerations. Furthermore, we will highlight key advancements and novel approaches in the field, including the incorporation of nonlinear optical effects for enhanced functionality.

Numerous other research papers have been published that either reviewed [59,60] or proposed [61,62] optical memory structures, including FFs. It is evident that over time, improvements have been observed in both the simulation results and the simulation tools employed, and these aspects will be elaborated upon in the conclusion.

By examining the latest research findings and technological advancements, this paper aims to contribute to the understanding of PC FFs as crucial components in the development of all-optical memory systems. The potential benefits of these memory components, such as high-speed operation, low power consumption, and compact integration, make them promising candidates for future optical computing applications.

## 2. Fundamental Properties of Photonic Crystals in All-Optical Memory

PCs are periodic structures that exhibit a unique ability to manipulate and control the flow of light. They are composed of materials with alternating regions of high and low refractive index, forming a periodic array. This periodicity gives rise to a PBG, a range of frequencies wherein the propagation of light is forbidden, for one, two or any number of polarizations. This property makes PC particularly useful for controlling and manipulating light waves in various applications, including optical memory components such as FF.

All-optical memory components utilize materials that can change their optical properties in response to external stimuli such as heat or light; these changes allow for the encoding and storage of information in the form of light signals.

PCs can be employed in optical memory systems in different ways; one common approach is to use the PBG property of the crystal to create a structure called a “resonator” or “cavity”.

A PC resonator consists of a defect within the periodic crystal lattice that introduces localized states within the PBG. These localized states can trap and confine light within the defect region. By carefully engineering the properties of the defect and the surrounding crystal structure, it is possible to create resonant modes at specific frequencies within the bandgap. To encode information, the resonator can be designed to have two or more distinct resonant modes. These modes correspond to different energy levels or states. By selectively exciting or suppressing certain modes, information can be stored as binary data (0 s and 1 s). For example, the presence or absence of a particular mode can represent a digital bit.

The advantage of using PCs in optical memory lies in their ability to confine and control light within a small volume. This confinement leads to enhanced light–matter interactions, enabling efficient read and write operations. Additionally, the PBG property ensures that the stored information is protected from external disturbances and scattering, improving the stability and reliability of the memory system.

Presented in the paper are different implementations and designs of PC-based all-optical FFs; they vary by utilizing different materials, architectures, and techniques to try and achieve the best -performing device possible.

## 3. Fundamental Properties of All-Optical Flip-Flops

### 3.1. Operating Wavelength

The operating wavelength range in optical components is a very important parameter in terms of compatibility with industry standards, for example, working at the C-band range, between 1540 nm and 1570 nm, means more compatibility with the current leading technology in the industry, the CMOS.

The operating wavelength is the optimal wavelength that is derived from the operating wavelength range.

### 3.2. Switching Speed

Switching speed refers to the time it takes for the optical memory device, for example, an FF, to transition between its two stable states. Switching speed is a critical parameter for optical FFs as it determines how quickly the stored information can be updated or accessed. Faster switching speeds allow for rapid data processing, high-speed communication, and efficient information storage and retrieval. The switching speed of an optical FF is influenced by several factors; one key consideration is the choice of optical components, such as waveguides, optical switches, RRs, or modulators. Another consideration is the overall design and architecture of the device, as factors such as the physical layout, the connectivity of the optical components, and signal routing mechanisms play a role in determining the overall speed of state transitions. Optimized architectures that minimize signal propagation delays and maximize light–matter interactions can help achieve faster switching speeds.

### 3.3. Contrast Ratio

The contrast ratio (CR) refers to the ratio between the intensity or power of the output signal in one state (such as a logical “1”) compared to the intensity or power of the output signal in the opposite state (such as a logical “0”). It measures the distinction or difference between the two states and is an important parameter for the performance and reliability of optical FFs. A high contrast ratio is desirable in optical components and FFs in particular, as it ensures clear differentiation between the different states, minimizing errors and improving the accuracy of information storage and retrieval. A higher contrast ratio translates to a better distinction between logical “1” and “0” states, enabling more reliable and accurate data processing. The CR is given as:

(1) $$\mathrm{CR}=10\log\frac{p1}{p0}$$

where p1 and p0 are the output power levels of logic 1 and logic 0, respectively.

## 4. Different Flip-Flop Variations and Methods of Design

### 4.1. The D Flip-Flop

**Figure 1:**
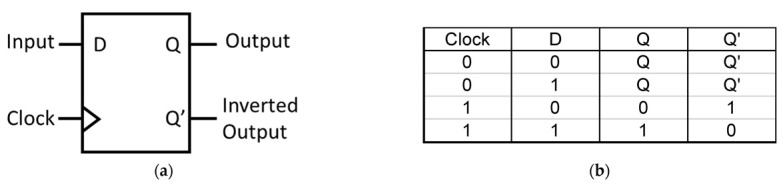
(**a**) Block diagram and (**b**) truth table of a D FF.

In regular use cases, an FF is a sequential logic circuit that can store and manipulate binary data. The D FF, also known as a data FF, is the simplest type of FF. It has a single data input (D) and two outputs: Q (the stored value) and Q’ (the complement of the stored value), as seen in Figure 1.

The stored value (Q) is updated synchronously with the CLK signal. When the CLK signal transitions from a high logic state to a low logic state, or the other way around, depending on the implementation, the current value of the D input is transferred to the output. In today’s system-on-chip (Soc) designs, the D FF is most commonly used for delay, synchronization, and memory applications.

In 2020, Saranya et al. presented a design of an all-optical clocked D FF for 1.72 Tb/s optical computing. The device is composed of 20 × 11 silicon and chalcogenide glass rods in a square lattice PC structure, with the refractive index of 3.46 and 3.1, respectively. The lattice constant a is 547 nm and the radius of the rods is 0.2a. The PBG for the silicon rods is 1327–1917 nm, and for the chalcogenide glass is 1238–1724 nm. The operating wavelength for the proposed structure is 1550 nm.

**Figure 2:**
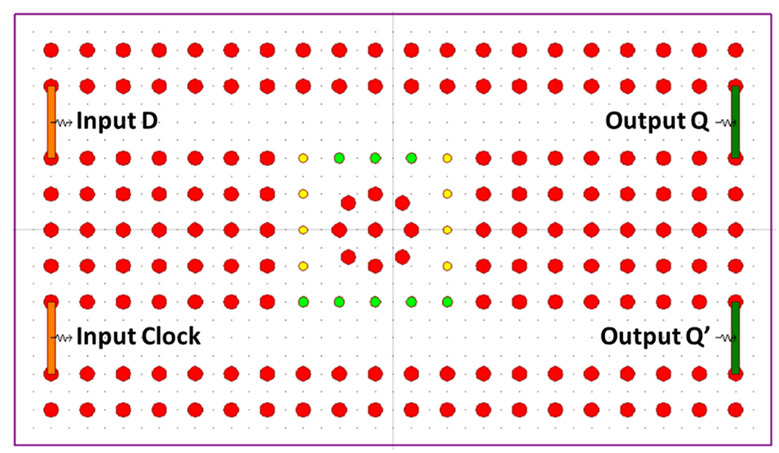
Schematic structure of the proposed D FF [63].

Using silicon as the refractive index, the proposed D FF has two waveguides that provide input at the first part of the design and output at the second part of the design. Connecting the two parts of these waveguides is an RR whose inner part is circular, and the outer is square. The component comprises a pair of inputs, namely, Clk and D, alongside two corresponding complementary outputs labeled as Q and Q’. The coupling rods, tinted in green, serve the purpose of linking the light signal and possess a radius of 0.14a. Conversely, the scattering rods, depicted in yellow, are designed with a radius of 0.12a to prevent undesired light leakage. Figure 2 displays a schematic of the proposed structure.

A Gaussian pulse is applied to the input with an operating wavelength of 1550 nm and a phase shift to get the desired outputs (Q, Q′). The contrast ratios for the output ports Q and Q′ are calculated using Equation (1), and determined to be 11.13 dB and 3.353 dB, respectively.

**Figure 3:**
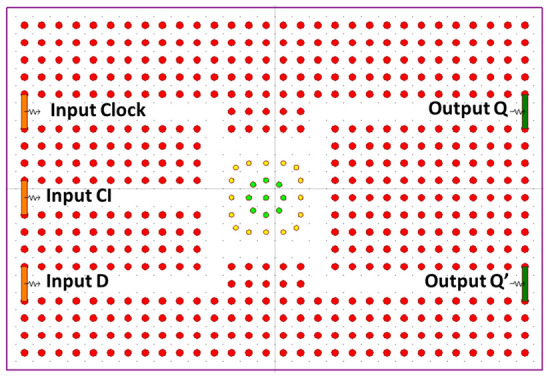
Schematic diagram of modified D FF [63].

The fundamental configuration under consideration is evaluated utilizing both silicon and chalcogenide glass as potential refractive indexes. After careful examination, it was determined that employing chalcogenide glass yielded a more favorable contrast ratio. In this study, a 30 × 20 μm square lattice 2D PC is meticulously devised. The lattice constant a is set to 600 nm, and the rod radius is fixed at 0.2a. Specifically focusing on the chalcogenide glass refractive index, the proposed structure is adapted through the incorporation of bend waveguides. These waveguides are designed to manipulate light signals without causing any adverse impact on the optical properties of field propagation. The revised configuration introduces three inputs, Clk, CI, and D, and establishes two outputs, Q and Q’. Within the design lies a square resonator, with its innermost rods possessing a radius of 0.16a, indicated in green, surrounded by an outer circle with a radius of 0.14a, depicted in yellow. The resonant wavelength characterizing the modified structure is recorded at 1550 nm. The altered arrangement is visually depicted in Figure 3.

**Figure 4:**
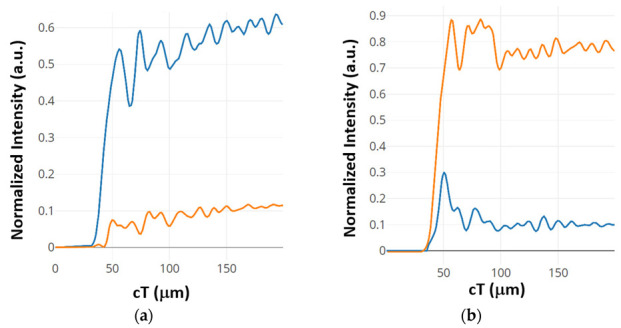
Normalized intensity of D FF at (**a**) port Q’ and (**b**) port Q [63].

The contrast ratios are calculated to be 8.75 dB and 7.63 dB for the outputs Q and Q’, respectively. Both silicon and chalcogenide glass were employed as the refractive index to analyze the structure. The output power of the structure is shown in Figure 4. The blue colored line describes the state where D = 0, Clk = 1 and CI = 1. The orange colored line describes the state where D = 1, Clk = 1 and CI = 0.

**Figure 5:**
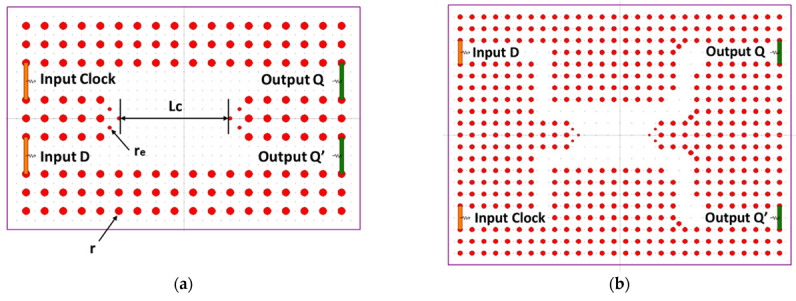
Structure of (**a**) basic and (**b**) modified clocked D FF design [64].

In 2017, Shaik et al. proposed a PC-based D FF scheme utilizing an MMI. This design incorporated a central MMI waveguide to facilitate the interference of the input light, while input and output ports were positioned at both ends of the MMI waveguide. Through an array of simulations, they demonstrated a contrast ratio of 9.63 dB and 5.84 dB at the Q and Q’ outputs, respectively. Furthermore, the response time was measured to be under 0.29 picoseconds. The fundamental configuration of this design is depicted in Figure 5a. In order to achieve a delayed output, a strategic approach was employed where the input and output waveguides were extended using sharp and smooth bend waveguides, respectively. This modification aimed to enhance the delay characteristics of the structure, aligning with the primary objective of the D FF concept. The designed clocked D flip-flop (D FF) architecture comprises an arrangement of 11 × 18 silicon rods organized in a square lattice configuration set against an air background, as illustrated in Figure 5b. The structure is outfitted with two input ports, D and Clk, along with two output ports, Q and Q’. During optimization, the rod radius was fine-tuned to 0.2a, except for the edge rods situated within the MMI waveguide, which were adjusted to a radius of 0.1a. The lattice constant used for this arrangement measures 600 nm.

The PBG was calculated using the PWE method; the wavelength was extracted from the PBG as 1430–2120 nm, and the operating wavelength was chosen to be 1550 nm. For the envisioned configurations, output transmissions falling below 0.25 and exceeding 0.75 are designated as logic 0 and logic 1, respectively.

**Figure 6:**
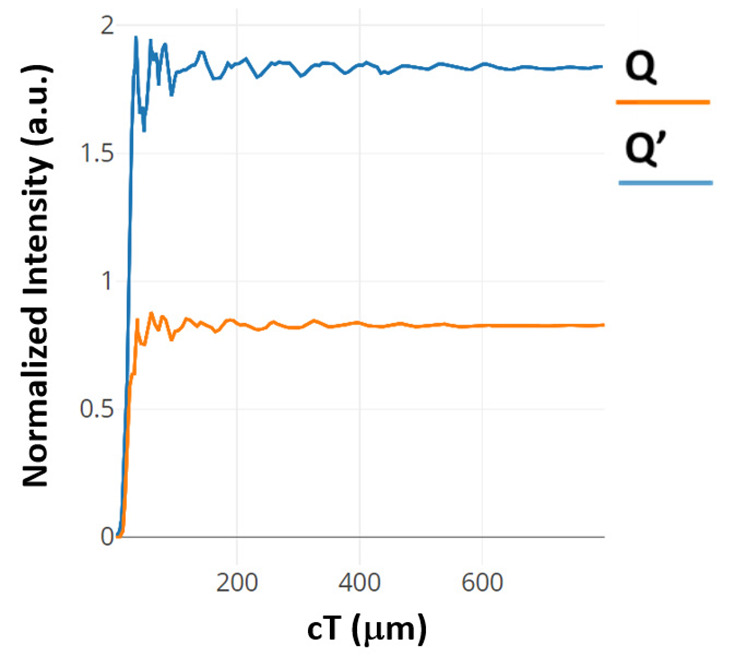
Normalized intensity at the output of the basic clocked D FF at port Q and Q’ [64].

The time taken for the output power at port Q to transition from 0 to 90% of the average power is comprised of two constituents. The first component corresponds to the transmission delay, denoted as t1, quantified at 0.049 ps. The second facet involves the time needed to traverse from 10 to 90% of the average power, represented as t2, calculated to be 0.056 ps. These components are illustrated in Figure 6. Given the linear properties of the proposed structure’s materials, the falling time extends from the average power to 10% of it, approximating t2. Consequently, the response time is determined to be 0.224 ps. Similar computations derived from the temporal evolution curve at port Q’, also presented in Figure 6, reveal a response time of 0.28 ps.

The proposed configuration has been enhanced through the implementation of L-bend waveguides, depicted in Figure 5b, with the primary intention of augmenting delay or response time. On the input side, sharp L-bends are integrated to manipulate the light’s propagation direction, while preserving its optical properties. Smooth L-bends are established at the output side using point defects, intended to introduce a delay to the input signal and diminish both electrical and optical interactions amid the waveguides. This is achieved through the utilization of a point defect located at the edge of the smooth L-bend, effectively acting as a cavity, and contributing to delay enhancement.

**Figure 7:**
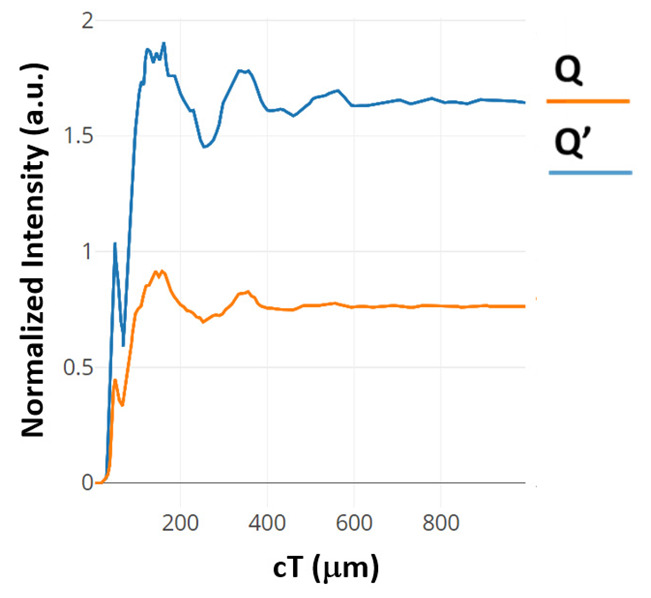
Time evolving curves of the modified clocked D FF at port Q and Q’ [64].

The response time of this structure, as deduced from the time-evolution curves illustrated in Figure 7 for ports Q and Q’, registers at 0.76 and 0.764 picoseconds, respectively. The CR has also been evaluated using output power levels at ports Q and Q’, yielding values of 9.92 dB and 5.27 dB, respectively.

**Figure 8:**
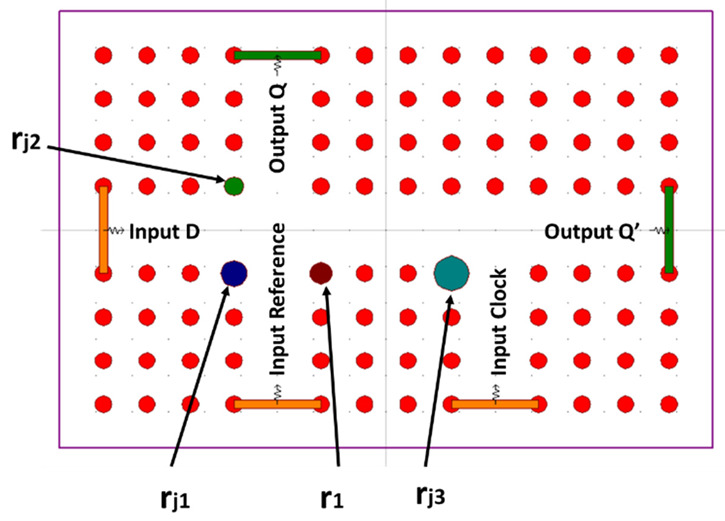
Structure of proposed PC based all-optical D FF [65].

In 2020, Rao et al. introduced an all-optical D FF design utilizing photonic crystal (PC) waveguides, strategically developed for optical computing and networking applications. This specific architecture was formulated through the implementation of T-shaped waveguides within a square lattice PC. Notably, the utilization of non-linear materials was deliberately avoided. The configuration featured silicon rods with a radius measuring 0.19a and a lattice constant of 0.6 µm. To facilitate light propagation, defects were skillfully introduced into the design, serving as waveguides, as visually depicted in Figure 8. At both junctions, specific rod radii were employed: rj1 at 0.18 µm, rj2 at 0.132 µm, rj3 at 0.24 µm, and the reflecting rod r1 at 0.15 µm. These variations were strategically incorporated to prevent undesirable back reflections into an unused input port. For instance, when the input D is set to logic 0, and both the reference and CLK inputs are at logic 1, the resultant light signal is directed away from input port D, favoring its propagation towards the output port Q’.

The waveguide situated on the left-hand side of the configuration serves as the input port designated for D. Positioned at the bottom of the structure are two vertical waveguides, functioning respectively as the reference input port R and the CLK input. Adjacent to the right edge of the design is a horizontal waveguide, allocated for use as the output port Q’. Correspondingly, the topmost position features a vertical waveguide designated as the output port Q.

**Figure 9:**
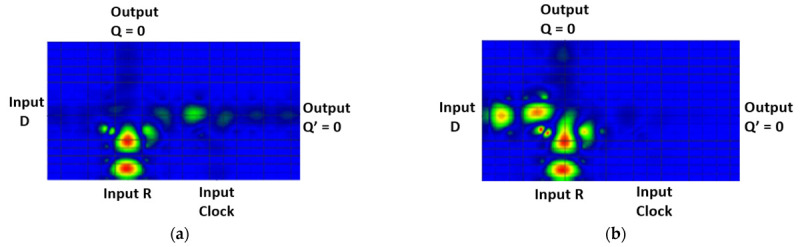

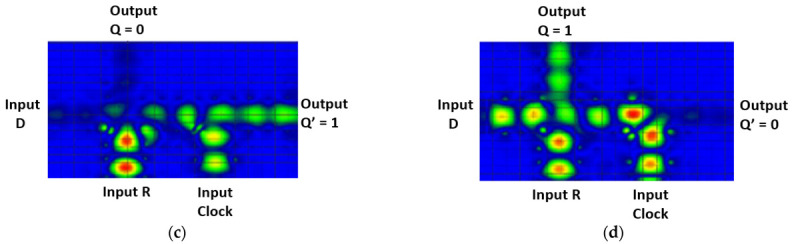
Propagation of light through the all-optical D FF for input conditions: (**a**) D = 0, CLK = 0; (**b**) D = 1, CLK = 0; (**c**) D = 0, CLK = 1; and (**d**) D = 1, CLK = 1 [65].

The operation of the all-optical D flip-flop is meticulously simulated and confirmed using OptiFDTD software, provided by OptiWave, based in Canada [66]. The proposed design employs a beam-interference principle, capitalizing on a continuous wave (CW) light source with a wavelength of 1550 nm. Through simulations, the intended functionality has been successfully showcased, demonstrating all four operational states of the device. Figure 9 shows the four operation states of the D FF; by designing specific waveguide path lengths, constructive and destructive interference are achieved, resulting in the desirable operation.

The CR has been calculated using Equation (1) to be 13.57 and 25 dB for outputs Q and Q’, respectively.

### 4.2. The SR Flip-Flop

**Figure 10:**
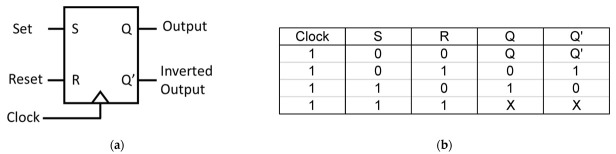
(**a**) Block diagram and (**b**) truth table of an SR FF.

The SR FF, also known as the set–reset FF, is a basic FF with two inputs, S (set) and R (reset), and two outputs, Q and Q’, as seen in Figure 10. The S and R inputs determine the FF state changes when the CLK signal transitions. When S = 1 and R = 0, the output is set (Q = 1) on the CLK edge. When S = 0 and R = 1, the output is reset (Q = 0). When both S and R are 0, the output remains in its current state. The SR FF is widely used for memory circuits, latches, and control systems.

In 2021, Hassan et al. presented an efficient and compact SR FF optical memory-based PC platform. The proposed design is based on two optical NOR logic gates which use two-dimensional PC with a square lattice of silicon dielectric rods in an air background. The lattice constant is 630 nm, and the radius of the silicon rods is equal to 0.2a; the PBG was calculated, and the operating wavelength range was extracted to be 1500–2172 nm and 851–868 nm.

**Figure 11:**
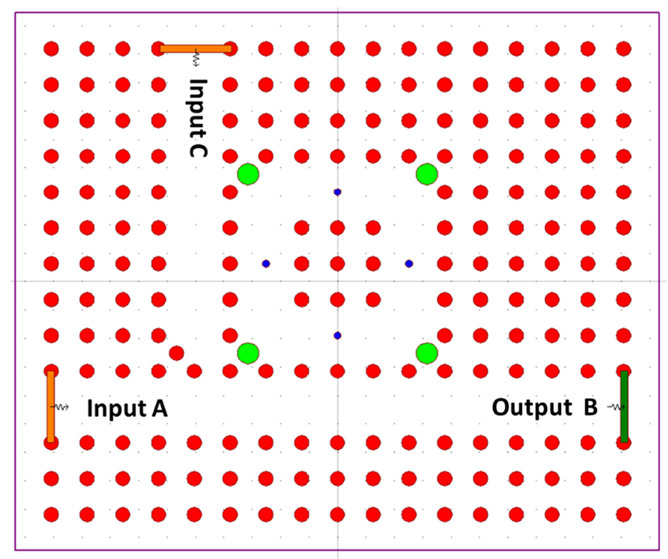
The proposed optical T-switch based on PC RR [67].

The optical memory SR FF employs optical NOR gates, constructed using optical T-type switches, PC RR, and an array of waveguides equipped with optical power drivers and Y-splitters. The optical NOR gate, designed within a 2D PC framework, is assembled by integrating two optical T-shaped switches. These switches consist of PC resonant rings along with T-type waveguides, which are formed through the introduction of specific defects into the structure of the 2D PC, as visually represented in Figure 11.

**Figure 12:**
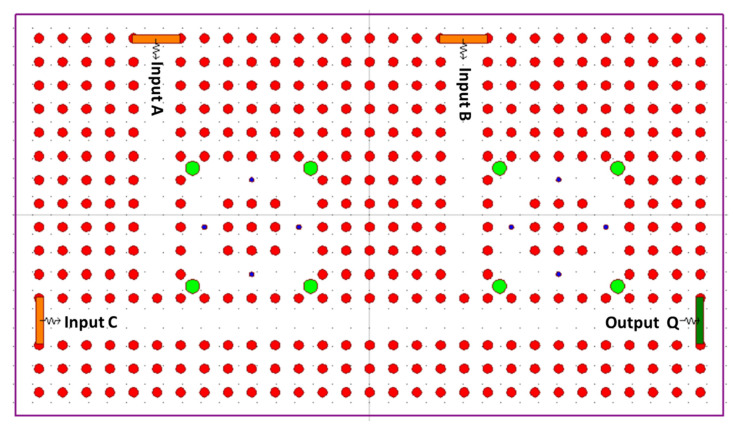
The proposed optical logic NOR gate [67].

The switch integrates three distinct ports: input port A, pump port C, and output port B. Connecting these ports are PC RR, and at the corners of this connection, four additional silicon rods, each with a radius of 0.3a, have been introduced. These rods are depicted in green and are strategically positioned to prevent the reflection of light waves. In addition, four more rods, shown in blue, and with a radius of 0.1a, have been incorporated. These blue rods serve the dual purpose of enhancing the interaction between waves and materials within the PC RR, as well as elevating the Q-factor. This elevated Q-factor significantly influences the energy stored within the proposed PC RR. The respective blue and green colors of these rods are visually evident in Figure 11. The optical NOR gate incorporates a pair of consecutive optical T-shaped switches, employing uniform materials, lattice constants, and rod radii. These two optical T-shaped switches are designed with three ports. The initial port, referred to as the BIAS port, is situated between C and Q. It has been established by selectively removing silicon rods to facilitate the uninhibited propagation of the data signal. The remaining two input ports, labeled A and B, are introduced to establish a linkage between the data signal and the PC RR, as depicted in Figure 12.

The optical wave, characterized by a wavelength of 1600 nm, penetrates the arrangement through the C port, while the determination of the NOR gate’s output state is governed by the inputs from the A and B ports. When either or both of the logic inputs, A and B, are in a logic 1 state, the output Q will assume a logic 0 state. Conversely, if both A and B are not in a logic 1 state, Q will transition to a logic 1 state.

**Figure 13:**
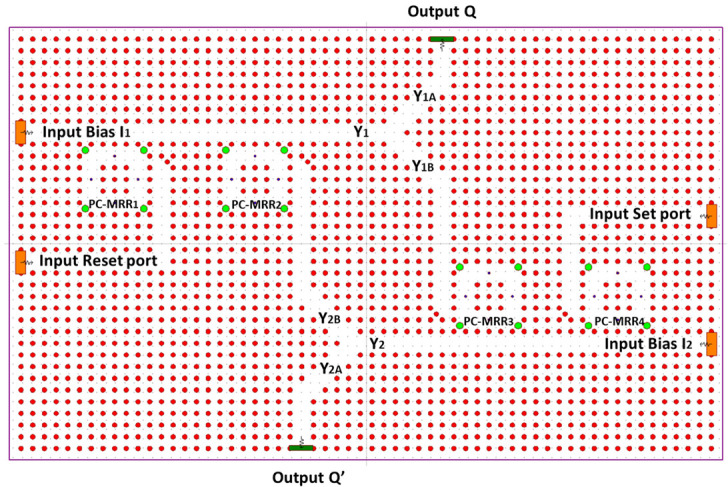
The proposed structure of optical SR FF based on 2D PC [67].

The proposed optical memory SR flip-flop is depicted in Figure 13. This design encompasses two input ports, namely, SET and RESET, supplemented by additional input bias ports I1 and I2, along with two output ports, Q and Q’. The optical SR flip-flop configuration comprises two optical NOR logic gates utilizing four PC RR, namely, PC-MRR1, PC-MRR2, PC-MRR3, and PC-MRR4. These gates are interconnected by means of optical T-shaped waveguides that are linked to two Y-splitters. The division of optical power from the waveguides is achieved through optical PC Y-splitters.

With a transmission efficiency of 94% and a 50:50 splitting ratio, the optical power splitter within the photonic crystal attains a balanced power distribution, resulting in each half receiving 47% of the power flow from the main waveguide. The precision of optical bends and junctions plays a pivotal role in designing the PC power splitter. The main waveguide channel Y1 partitions the optical signal from the bias port I1 into two channels, denoted as Y1A and Y1B. Y1A directs the optical signal toward port Q, while Y1B serves as feedback for NOR2. Similarly, Y2 divides the optical signal from bias port I2 into two channels, Y2A and Y2B. Y2A steers the optical signal toward port Q’, and Y2B functions as feedback for NOR1, as illustrated in Figure 13. The operational wavelength for optical ports I1 and SET is set at 1600 nm, whereas for optical ports I2 and RESET, it is established at 1580 nm.

**Figure 14:**
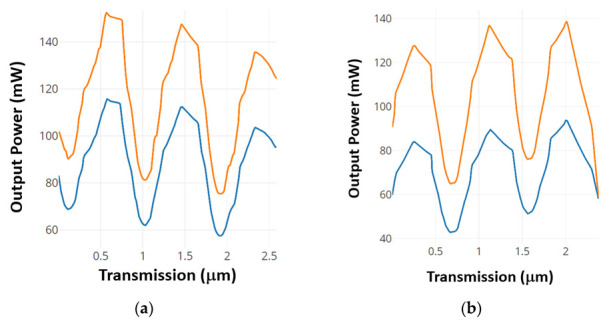
The output power (**a**) at port Q and (**b**) at port Q’ [67].

The optical input power for each input port at the active state is equal to 150 mW; the power is spread equally through the activated input ports. The proposed optical SR FF has four states and one undefined state. The set case sets the output Q to a logic 1, the reset case sets the output Q to a logic 0, and there are two no-change cases at which the output state is memorized. The states are demonstrated in Figure 14. Figure 14a describes the set case in orange color and the no-change state that occurs after in blue color. Figure 14b describes the reset case in orange color and the no-change state that occurs after in blue color.

As depicted in Figure 14, it becomes evident that the output power does not converge to a constant value, but rather exhibits oscillations. The power span corresponding to logic 0 ranges from 0 to 30 mW, while for logic 1, it spans 150 to 160 mW. Furthermore, the CR can be quantified as 4.77 dB for logic 0 and 6.99 dB for logic 1.

**Figure 15:**
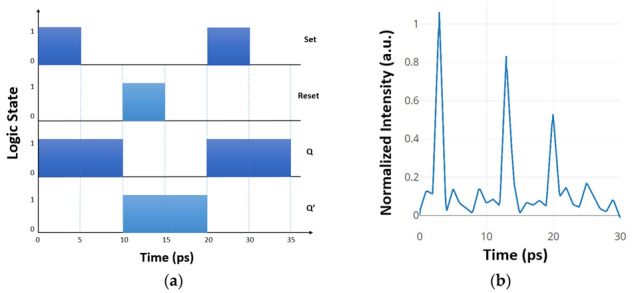
(**a**) Timing diagram for different SR FF states, (**b**) normalized intensity at the output of the SR FF [67].

Figure 15a concisely presents a timing diagram encompassing a sequence of events. Simulation outcomes indicate that by manipulating the input states of the SR FF, the rise and fall response times for the proposed SR FF memory correspondingly amount to 3 ps and 1 ps, as visually demonstrated in Figure 15b. Additionally, the output response time aligns with 1.2 ps, and the switching rate attains a notable 133 GHz.

In 2018, Zamanian-Dehkordi et al. introduced an all-optical RS FF utilizing nonlinear PC configurations. This innovative design capitalizes on the nonlinear Kerr effect within the PC framework. The proposed architecture is composed of a central segment and two optical switches. The central section encompasses two interconnected resonant cavities, each resonating at distinct wavelengths: 1586 nm and 1620 nm. The fundamental PC structure is in a square lattice configuration, featuring dielectric rods immersed in an air medium. The dielectric rods possess a refractive index of 3.5, and the lattice constant a measures 575 nm. For switch 1 and switch 2, the radii of the rods are 0.2227a and 0.237a, respectively.

Using the PWE method, two PBG were found for each switch at TM mode; the operating wavelength range for switch 1 is at 1489–2186 nm and 1058–1134 nm, and the operating wavelength range for switch 2 is at 1562–2255 nm and 1065–1190 nm.

**Figure 16:**
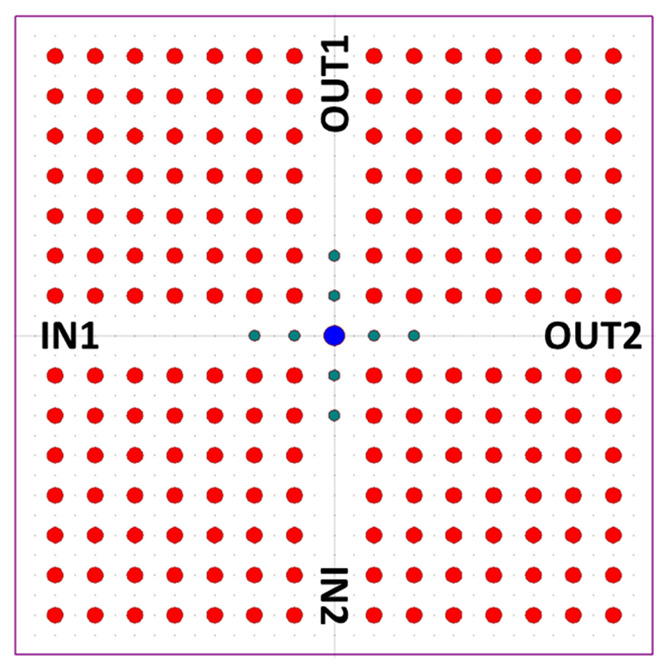
The core section of the proposed device [68].

The core section is shown in Figure 16 and is composed of two cross-connected resonant cavities with a nonlinear elliptical defect shown in blue color. IN1 and IN2 are inputs and Q and Q’ are the output ports.

The rods constituting the foundational structure, illustrated in gray, possess a refractive index of 3.5. The red rods are characterized by a lattice constant of 575 nm and a radius equivalent to 0.15a. The elliptical defects, marked by radii of 0.29a and 0.31a, have a nonlinear coefficient of $9\times 10^{-17}\frac{m^{2}}{W}$. Simulations reveal the absence of a PBG in the TM mode, while two distinct PBGs emerge in the TE mode: one spans 1389–2061 nm and the other occupies 783–805 nm. Upon introducing varying wavelengths into IN1 and IN2, it becomes evident that the cavities exhibit resonant modes at 1620 and 1586 nm. These resonant wavelengths enable the emission of light from the core section.

**Figure 17:**
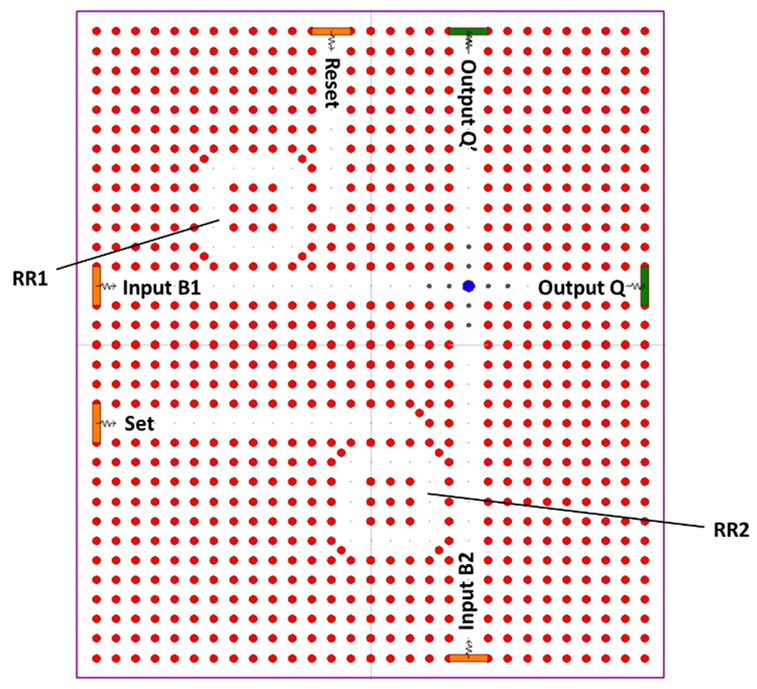
The final structure of the proposed RS FF [68].

The final design for the RS FF is shown in Figure 17; the device has two input ports, set and reset, two bias ports B1 and B2, and two output ports Q and Q’. RR1 and RR2 symbolize the representation of two RRs for each of the switches. There are five different states that were simulated.

**Figure 18:**
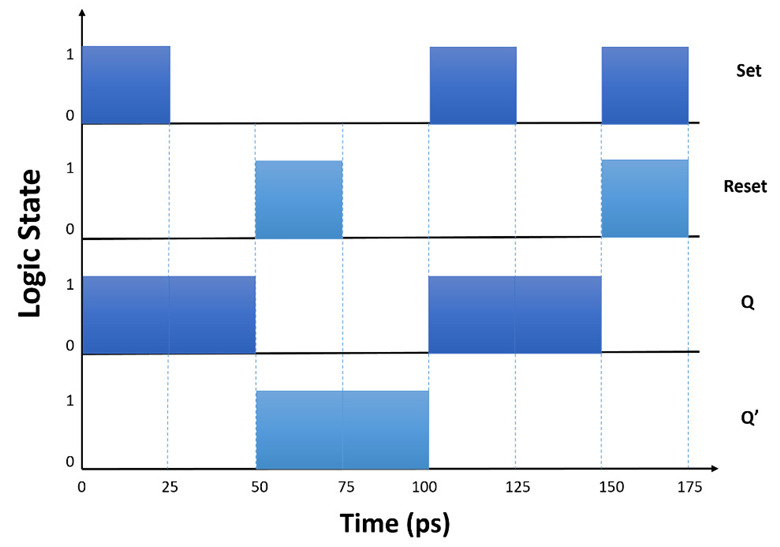
Time response of the structure with reset and set inputs [68].

Figure 18 illustrates the time response diagrams, showcasing a continuous presentation of all simulated states of the FF. The optical power applied to the input ports is set at 100 mW. Modification in the input logic states results in a rise time of 3.1 ps for Q and 3 ps for Q’, correspondingly. The fall time for these transitions is 1 ps. Rise and fall times denote the intervals required to shift between the amplitude values of 10% and 90%, and vice versa, respectively. Consequently, the maximum response time is recorded at 3.1 ps, while the switching speed registers 320 GHz. The normalized power margins for logic 1 and 0 are obtained at 65% and 7%, respectively.

In this diagram the output power does not converge to a constant value, it oscillates. This issue is mainly due to reflection at the core section of the resonant cavity and due to the nature of the nonlinear resonant cavity. The suggested design enhances the switching speed at the expense of optical power, yet the operational efficacy of the proposed structures demands a higher optical power compared to other suggested designs.

In 2022, Soma et al. proposed a design of 2D PC-based ultra-compact optical RS FF. The FF is designed using two NOR gates, PC waveguides, four silicon RRs, four input ports, and two output ports. The structure consists of hexagonal silicon rods in an air background with a lattice constant a of 630 nm, rod radius of 0.2a, and operating wavelength of 1550 nm.

**Figure 19:**
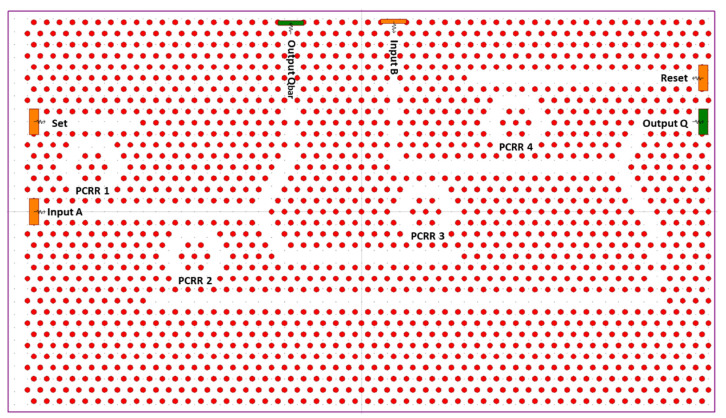
Structure of proposed RS FF [69].

Figure 19 shows the proposed structure of the RS FF constructed using a 2D hexagonal lattice PC; the lattice structure is constructed using 55 × 34 silicon rods with air as a background. The PBG is calculated using the PWE method, and the operating wavelength range is extracted from the PGB to be 1316–1978 nm.

The proposed configuration involves the interconnection of two NOR gates. The initial NOR gate is responsible for receiving the reset input and the reference input “B”. It is created by utilizing two ring resonators (PCRR3 and PCRR4) along with a T-type waveguide. The outcome of the first NOR gate is divided into two using Y splitter ports. One branch is directed towards output port Q, while the other serves as feedback to the input of the NOR gate.

The second NOR gate is designed to handle the set input and the reference input a. This is achieved by employing two different ring resonators (PCRR1 and PCRR2) in combination with a T-type waveguide. Similar to the first NOR gate, the output of the second NOR gate is divided into two using Y splitter ports. One of these outputs is collected from output port Q’, and the other is routed back to the input of the first NOR gate as feedback.

The outputs from Q and Q’ of each NOR gate are also looped back to the input of both NOR gates through Y splitter connections. The operational wavelength for the optical source applied to the inputs is 1550 nm, and the intensity of the optical signal used is 1 arbitrary unit (a.u.). If the intensity of the optical signal surpasses 0.5 a.u., it is interpreted as logic 1, while a signal with an intensity below 0.5 a.u. is considered logic 0.

The operational characteristics of the newly proposed FF are evaluated across various states using the FDTD method. From the simulation outcomes, it is determined that the contrast ratio at the output ports Q and “Qbar” measures 8.7 dB and 4 dB, respectively. The response time for Q is approximately 1.2 ps, while for “Qbar” it is around 2.6 ps.

Additionally, the physical dimensions of the proposed FF are smaller, spanning 28 μm by 28 μm, when compared to what is reported in the previous literature, as outlined in the paper. This compact size is complemented by its fast-switching frequency.

### 4.3. The T Flip-Flop

**Figure 20:**
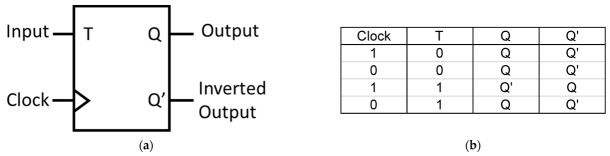
(**a**) Block diagram and (**b**) truth table of a T FF.

The T FF, also known as a toggle FF, is an extension of the D FF. It has a single input called T and two outputs Q and Q’, as shown in Figure 20. The T input determines whether the FF state toggles or remains unchanged when the CLK signal transitions. The T FF is often used for frequency division, counters, and control circuitry.

In 2021, M. Valliammai et al. offered a new design for an all-optical chalcogenide T FF utilizing a PC waveguide. The structure of the FF comprises chalcogenide rods arranged in a square lattice on an air substrate. The lattice constant a is maintained at 600 nm, the rod radius is set to 0.19a, the small rod’s radius “re” is equal to 0.1a, and the operating wavelength is 1550 nm.

**Figure 21:**
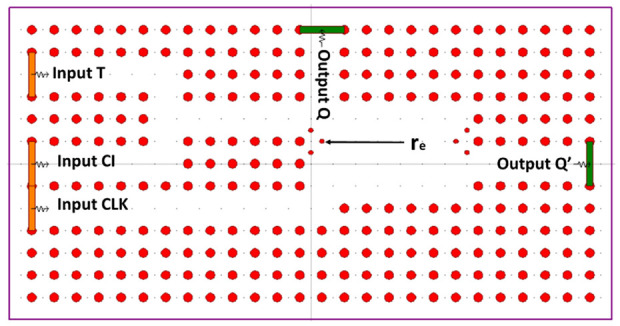
Light propagation in the “11” state of the T FF [70].

Figure 21 shows the structure of the T FF, which is formed by combining an XOR gate with a D FF. This construction involves incorporating an array of chalcogenide rods arranged in a square lattice, consisting of 26 × 13 rods, exposed to an air substrate.

The proposed structure consists of three input ports, T, Cl (control input logic), and CLK, as well as two output ports, Q and Q’. To confirm the toggle logic operation, the actual input bits are energized in the form of a light beam through the T input port. Specifically, the Cl input port represents the former state and appears at the output of the T FF, represented as Q. The CLK input port is activated with logical 1. The XOR receives inputs from the Cl and T ports to create logic values for “D”. The D FF section takes inputs from the CLK port and the output of the XOR section is utilized as an input signal. The implementation of a clocked D FF introduces a delay or acts as a buffer for its input signal.

**Figure 22:**
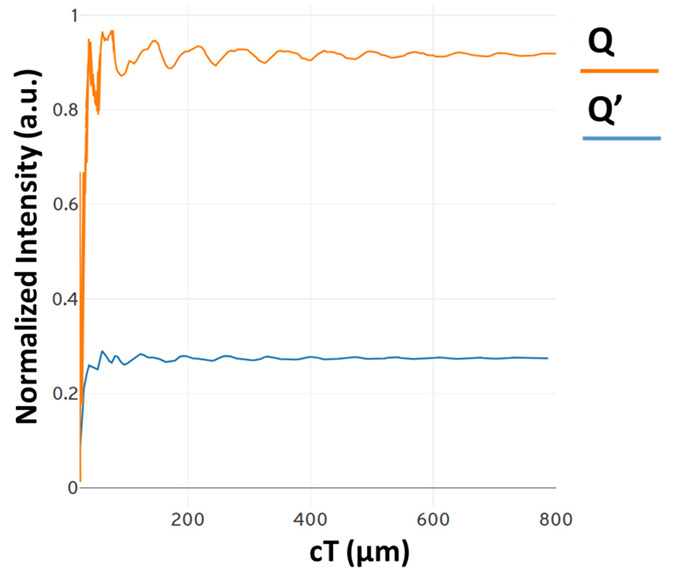
Power levels measured at ports Q and Q’ [70].

In Figure 22, the contrast ratio is depicted as a measure to illustrate the power level difference between the optical logic 0 and logic 1 states. The power level P0, which varies below 0.28, represents the optical logic 0 state through the Q port. Similarly, the power level P1, above the value of 0.89, signifies the optical logic 0 state through the Q’ port.

The effectiveness of this design is evaluated through mathematical analysis using the FDTD technique. The use of chalcogenide glass material proves beneficial in achieving a high contrast ratio, which enables clear differentiation between the logic 0 and logic 1 states.

### 4.4. The JK Flip-Flop

**Figure 23:**
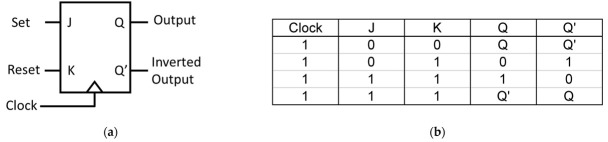
(**a**) Block diagram and (**b**) truth table of a JK FF.

The JK FF is an extension of the SR FF; it has two inputs, J (set) and K (reset), and two outputs, Q and Q’, as shown in Figure 23. The J and K inputs determine how the FF state changes when the CLK signal transitions. When J = 1 and K = 0, the output is set (Q = 1) on the CLK edge. When J = 0 and K = 1, the output is reset (Q = 0). When both J and K are 1, the output toggles (Q = Q’ or Q’ = Q).

In 2021, a clocked JK FF design was proposed by K. Rao et al. using an advanced air-hole type PC. The structure of the FF is based on the principles of MMI. The proposed JK FF structure consists of an arrangement of 15 × 21 poles organized in a square grid within an air medium. The lattice constant a is 500 nm, while the rod radius is set at 0.5a. The operating wavelength for this design is chosen as 1650 nm. The coupling length, denoted as Lc, of the MMI waveguide is derived from the scattering characteristics of the waveguide, and is specifically set to 8.2a.

**Figure 24:**
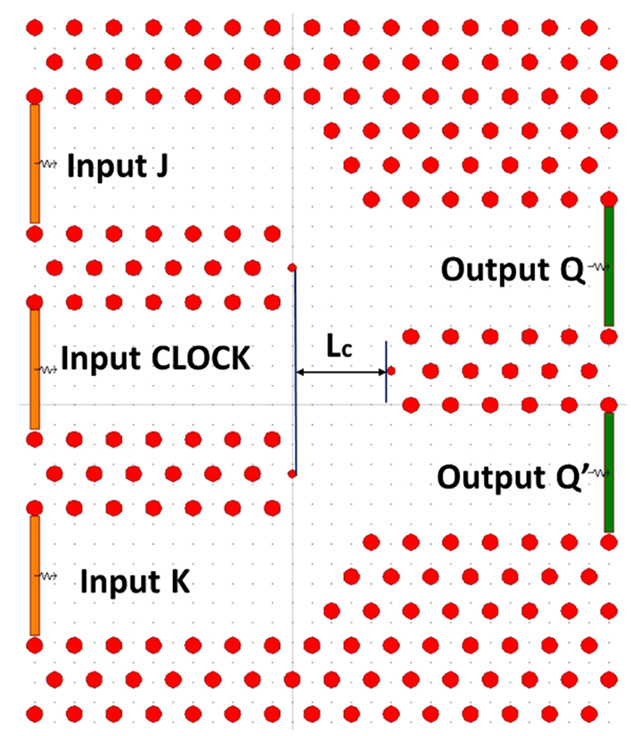
Structure of proposed clocked JK FF [71].

The structure of the JK FF, as depicted in Figure 24, comprises a waveguide or MMI region. The MMI area is designed to support a significant number of modes and is connected to input and output waveguides at its front and back ends. These additional waveguides facilitate the transmission and retrieval of light to and from the main MMI waveguide, enabling the operation of the JK FF.

The JK FF consists of three input ports, namely, J, K, and CLK, as well as two output ports, Q and Q’. The J and K ports are responsible for receiving the input bit patterns, while the CLK port is used for the positive-level triggered CLK input. The outputs, Q and Q’, represent the resulting binary values generated by the FF.

The photonic band structure is utilized to determine the PBG regions of the cross-section of the PC where the waveguide is constructed. The band structure and scattering behavior are determined using the PWE method.

The determination of the coupling length (Lc) in the system involves considering four possible guided modes: the essential mode, first-order mode, second-order mode, and third-order mode. The guided modes in the PC-based MMI waveguide are determined based on the working point, which depends on the working frequency. At the working point of a equivalent to 0.367, corresponding parameters for the guided modes are obtained and recorded in Table 1, where b is the generated constant of the requested mode.

The coupling length (Lc) is determined by the essential mode and the second-order mode, ensuring the proper coupling of the three information signals. At this coupling length, the MMI waveguide’s output port receives more power, while the other output port receives lower or insignificant power. When a single information port is activated, a single image occurs at multiples of the beat length, which is approximated as the coupling length (Lc) in the structure. The coupling length is set at 8.2a, obtained from the spacing between the focal points of the extreme edge bars in the MMI waveguide multiplied by half of the rod radius.

**Figure 25:**
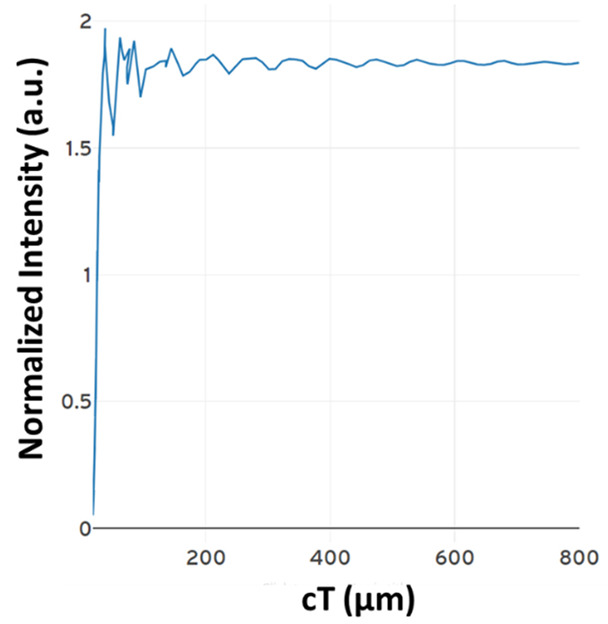
Response time of FF for change in output [71].

Figure 25 displays the response time of the JK FF for each test case.

Table 2 presents the final contrast ratio results for each output of the JK FF.

The results show that the contrast ratios at Q’ and Q are 8.657 and 6.24 dB, respectively. Additionally, the output time response is measured at 0.27 ps. The structure exhibits a very low response time. Its simplicity, high contrast ratio, power output, and quick response time make it highly suitable for integration into optical circuits.

## 5. Conclusions

This paper investigates various structures of all-optical FFs based on PC. The four types of FFs studied are D FF, SR FF, T FF, and JK FF. The key parameters evaluated include response time, contrast ratio, footprint, operating wavelength range, and operating wavelength, as seen in Table 3, which summarizes all the structures discussed in this paper. The contrast ratio range observed in these structures is 6.91–16.68 dB, ensuring reliable differentiation between logic 0 and logic 1 states. The operating wavelength range is 1550–1650 nm, falling within the C and L bands, crucial for optical communication systems like telecommunications networks and fiber optic links. The footprint range for the all-optical FFs is 38.85–836 μm^2^, which enables it to be integrated on a large-scale all-optical chip. The response time range is 0.063–3.1 picoseconds. The all-optical FFs’ structures are designed using RRs, waveguides, Y-splitters, and MMIs, employing PC technology and leveraging the optical interference effect. Different lattice types, square or hexagonal, and with and without non-linear materials, are used. Point defects, like scattering rods or coupling rods, are introduced to achieve the desired FF operation.

In a study published in 1999 by V. Stojanovic et al., an analysis of FFs was conducted using a set of rules to ensure fair and realistic comparisons between high-speed FFs built using different architectures [72]. The findings indicate that the response time of these FFs falls within the bracket of 180 to 630 picoseconds, which is considerably slower compared to the earlier mentioned range of 0.063 to 3.1 picoseconds. This substantial difference of 2 to 4 orders of magnitude underscores the remarkable capability of optical components, highlighting their superior potential in terms of operational speed when compared to conventional electrical components.

Different technologies have been proposed to make optical FFs; in a study published in 2005 by R. Clavero et al., an all-optical FF was proposed that is based on a single semiconductor optical amplifier-based Mach–Zehnder interferometer (SOA-MZI) [73]. Based on the simulations the authors performed, the response time of the structure is lower than 1 ns. In 2003, H.J.S Dorren et al. studied nonlinear polarization rotations in SOAs and how they can be applied to all-optical FFs [74]. The conclusion the paper presents is that an FF using their proposed mechanism can achieve a response time of around 100 picoseconds. A clear difference in speed can be observed when comparing the mentioned examples of all-optical FFs in different technologies to the structures in this review paper, which are all designed using PC technology. Based on the comparison made in this paper, a design can be chosen for different optical computing applications, depending on the preferable trade-off between its parameters. For example, devices with smaller footprints might be preferable to use in high-density integration applications, devices with high contrast ratios might be used in highly accurate optical computing, devices with specific operating wavelengths might be used in certain optical communication systems, etc. In conclusion, this paper highlights the significant advantages of optical FFs, making them a promising technology for advancing optical computing and optical memory systems.

## Figures and Tables

**Table 1:** Mode parameters of the waveguide at the working wavelength [71].

Mode	Propagation Constant at Wavelength of 1650 nm	Coupling Length (Lc) (μm)
Fundamental	0.36	21.2a
First Order	0.32	18.6a
Second Order	0.28	8.2a
Third Order	0.17	2.5a

**Table 2:** CR of the proposed clocked JK FF [71].

Q	Pout	CR	Q’	Pout	CR
0	0.216	8.657	21.2a	0.204	8.568
1	1.571		18.6a	1.498	

**Table 3:** Comparison of all works discussed in this paper.

Type of Structure	Mechanisms and Effects	Response Time (psec)	Contrast Ratio (dB)	Footprint (μm^2^)	Operating Wavelength (nm)	Year
Linear square lattice of Si * and chalcogenide glass ** rods in air [63]	Ring resonator, coupling rods and scattering rods	0.063	11.13 * 9.711 **	71.14	1550	2020
Linear square lattice of Si rods in air [64]	MMI, edge rods point defects	0.29	9.63	71.28	1550	2017
Linear square lattice of Si rods in air [65]	T-shaped waveguides	-	13.5	45.36	1550	2020
Linear square lattice of Si rods in air [67]	T-type switches, ring resonators and Y-splitters	1.2	6.99	836	1600 (SET) 1580 (RESET)	2021
Non-linear square lattice dielectric rods in air [68]	cross-connected resonant cavities and optical switches	3.1	9.68	361	1586 1620	2018
Linear hexagonal lattice Si rods in air [69]	Ring resonators and Y-splitters	1.2	8.7	784	1550	2022
Linear square lattice chalcogenide rods in air [70]	XOR gate, D FF, chalcogenide rods	0.16	16.68	38.85	1550	2021
Air holes in GaAs PC configuration [71]	MMI, scattering rods	0.27	8.657	78.75	1650	2021

* Parameters for Si rods. ** Parameters for chalcogenide glass rods.
